# Molecular Biology: The Shape of Food Allergenicity

**DOI:** 10.1289/ehp.113-a448

**Published:** 2005-07

**Authors:** Angela Spivey

Every year, food allergies cause about 30,000 visits to emergency rooms and an estimated 150 deaths. The culprits are known; only eight foods—milk, eggs, peanuts, tree nuts, fish, shellfish, soybeans, and wheat—cause 90% of all allergic food reactions. But why do those foods cause allergies while others don’t? A study in the January 2005 *Journal of Allergy and Clinical Immunology* suggests that the answer may lie partly in three-dimensional protein structures that are common to many different plants that cause allergies.

Scientists once thought that any protein could potentially become an allergen. In the current study, however, using a computer program to categorize 129 common plant food allergens, structural biologist John Jenkins of the British Institute of Food Research (IFR) and colleagues found that 65% of these proteins fell into just four structural families. The study used the protein families defined by Pfam, a database of protein structures housed at the Wellcome Trust Sanger Institute in the United Kingdom.

The results suggest that certain protein structures contribute to plants’ allergenicity, says coauthor Clare Mills, head of the allergy research team at IFR. The next step is finding out which structures contribute, and how they do so.

Some of these common structures may make a protein very stable, and thus hard to digest. For instance, one of the four dominant families identified in this study, the cupin family, has barrel-shaped sections (the family gets its name from *cupa*, a Latin word meaning “barrel”). This shape makes the proteins very stable, Mills says, adding, “If a protein is resistant to digestion, there’s more of it available for the immune antibodies to attack.”

The authors also analyzed surface structures in proteins that are cross-reactive. One family of proteins, the Bet v 1 homologues, showed an unusual conservation of surface shapes across different plants. The scientists studied the family closely to learn more about that conservation and how it underlies the allergic cross-reactivity between birch pollen and plant foods such as apples and celery.

“Generally, proteins change quite a lot on their surface when you go across different species,” Mills says. “But the Bet v 1 family is unusual. Although some of the amino acid residues changed [from the major birch pollen allergen Bet v 1 to the related apple allergen Mal d 1], the shape of the molecule was very much the same.”

According to Mills, the degree of change in the surface of the allergenic protein appears to correlate with the degree of allergic symptoms that people experience. A Bet v 1–related allergen, Api g 1, is found in celery, but its surface shape is altered more from Bet v 1 than that of the apple allergen. Similarly, people with birch pollen allergy can have cross-reactions to celery, but less often than they do to apples.

Mills and colleagues are conducting similar bioinformatics analysis of proteins in pollen and food allergens of animal origin to find out if these also show structural similarities. Although Mills says “it’s not a focus of our research to come up with an *in silico* method of looking for allergens,” she does say that categorizing proteins into structural families may also help in evaluating the potential allergenicity of proteins found in genetically modified foods. Many people are concerned that these engineered foods may introduce novel proteins that humans are unable to digest.

Stephen Howell, director of the Plant Sciences Institute at Iowa State University, agrees that the study suggests an additional parameter to be considered in evaluating novel proteins for allergenicity. Although new proteins introduced by genetic engineering are already tested extensively, he says that more knowledge can only help inform and improve that testing.

Richard Goodman, a research professor of food science and technology at the University of Nebraska–Lincoln, says that, in addition to bioinformatics tools, researchers may also need to use nuclear magnetic resonance spectroscopy or crystallography to examine tiny differences in surface structure to fully understand protein structures’ role in allergenicity. Allergy is a complicated condition that depends on the amount of allergen present in a food, how often a person has been exposed to it, how many immune cells react to the allergen, and how strongly the cells react. “But,” Goodman says, “this study does indicate that there might be more predictability to this than once thought.”

## Figures and Tables

**Figure:**
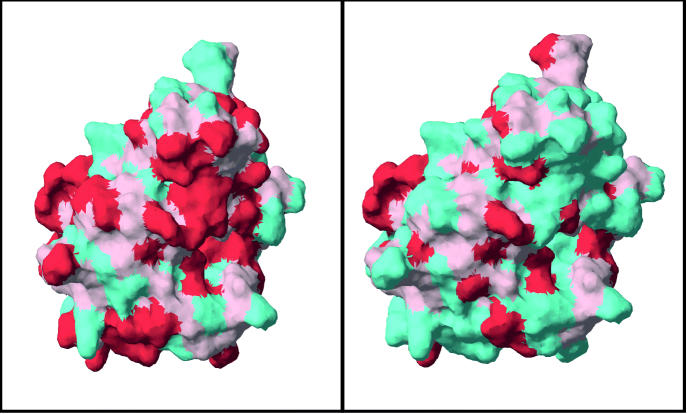
**Degrees of separation.** In the above comparison of proteins from apples (left) and celery (right) to that from birch pollen, red areas show where surface structure has been conserved across the proteins. Given these two proteins’ relative structural similarity to that of birch pollen, people allergic to birch pollen are more likely to also be allergic to apples than to celery.

